# Soluble High Mobility Group Box 1 (HMGB1) Is a Promising Biomarker for Prediction of Therapy Response and Prognosis in Advanced Lung Cancer Patients

**DOI:** 10.3390/diagnostics11020356

**Published:** 2021-02-20

**Authors:** Nikolaus A. Handke, Alexander B. A. Rupp, Nicolai Trimpop, Joachim von Pawel, Stefan Holdenrieder

**Affiliations:** 1 Department of Radiology, University Hospital Bonn, 53127 Bonn, Germany; Nikolaus.Handke@ukbonn.de; 2 Institute of Clinical Chemistry and Clinical Pharmacology, University Hospital Bonn, 53127 Bonn, Germany; rupp.alexander@googlemail.com (A.B.A.R.); Nicolai.Trimpop@ukbonn.de (N.T.); 3 Institute of Laboratory Medicine, German Heart Centre Munich, Technical University Munich, 80636 Munich, Germany; 4 Asklepios Lungen-Fachkliniken München-Gauting, 82131 Gauting, Germany; jvonpawel@yahoo.com

**Keywords:** lung cancer, biomarker, HMGB1, CYFRA 21-1, therapy monitoring, prognosis

## Abstract

Background: High mobility group box 1 protein (HMGB1) is known for its significant elevation in a multitude of tumors and benign diseases. In this study, we investigated the relevance of soluble HMGB1 for the prediction and monitoring of therapy response as well as the estimation of prognosis in advanced lung cancer. Materials and Methods: In a retrospective study, HMGB1 levels were assessed by an enzyme-linked immunosorbent assay (ELISA) in the sera of 96 patients with advanced lung cancer (79 non-small-cell lung carcinoma (NSCLC); 14 small cell lung carcinoma (SCLC), 3 Mesothelioma) prior to cycles 1, 2, and 3 of chemotherapy and correlated with radiological therapy response after 2 and 4 cycles as well as with overall survival. In addition, HMGB1 was compared with established tumor markers cytokeratin 19-fragments (CYFRA 21-1), carcinoembryonic antigen (CEA) and neuron specific enolase (NSE). Results: While pretherapeutic HMGB1 levels were not predictive or prognostically relevant in NSCLC patients, HMGB1 values prior to cycles 2 and 3 as well as kinetics from cycle 1 to 2 discriminated significantly between patients with good (remission and stable disease) and poor response (progression). Performance of HMGB1 in receiver operating characteristic (ROC) analyses of NSCLC patients, with areas under the curve (AUCs) of 0.690 at cycle 2 and 0.794 at cycle 3 as well as sensitivities of 34.4% and 37.5%, respectively, for progression at 90% specificity, was comparable with the best tumor-associated antigen CYFRA 21-1 (AUCs 0.719 and 0.799; sensitivities of 37.5% and 41.7%, respectively). Furthermore, high concentrations of HMGB1 at cycles 2 and 3 were associated with shorter overall survival in NSCLC patients. Conclusion: Soluble HMGB1 is a promising biomarker for prediction of therapy response and prognosis in advanced NSCLC patients.

## 1. Introduction

High mobility group (HMG) proteins were first isolated in 1973 by Ernest Johns, Clive Sanders and Graham Goodwin, who recognized the high mobility of these proteins in gel electrophoresis [1]. High mobility group box 1 protein (HMGB1) is a ubiquitous, highly conserved nuclear protein that can be detected intra- and extracellularly, orchestrating various site-specific functions. Intracellular HMGB1 plays an important role in the nucleus as a chaperone [2,3], whereas extracellular HMGB1 acts as a danger-associated molecular pattern (DAMP) in immunogenic cell death (ICD) [4,5,6]. Redox affinity of HMGB1 cysteine residues enables the molecule to organize inflammatory processes by switching between cytokine-inducing, chemoattractant activity or supposedly inactive status in the extracellular space [7,8]. High expression of HMGB1 in tumor cells mediates tumor progress through inhibition of apoptosis and elevated induction of cytoprotective autophagy [9,10,11]. Various studies report on the overexpression and increased release of HMGB1 in cancer, e.g., in non-small-cell lung carcinoma (NSCLC) [12,13,14,15], breast cancer [16], gastric cancer [17,18], hepatocellular carcinoma [19,20], pancreatic carcinoma [21], colorectal carcinoma [22,23] and lymphoma [24]. Predominantly undifferentiated tumors express high intracellular amounts of HMGB1 [25], hence raising interest in exploiting intra- and extracellular HMGB1 as biomarker for biochemical therapy evaluation in oncological patients.

With a global incidence of 2.1 million cases in 2018, lung cancer was the most frequent malignant neoplasm. Focusing on mortality rates in the same year, lung cancer caused most deaths with an estimated 1.8 million worldwide or 18% of all cancer deaths [26,27]. Current developments concerning higher life expectancy in industrial countries due to medical care as well as the population growth in less developed regions of the world with higher pollution rates indicate that the burden of lung cancer will rise and continue [28,29]. Due to late clinical manifestation, the majority of patients are diagnosed in advanced stages of disease with limited success of therapy and numerous comorbidities. Earlier detection and initiation of therapy improves prognosis [30]. 

Beside morphological changes, biochemical responses of the neoplasm to the treatment are one of the most important criteria concerning therapy effectiveness. At present, clinical, radiographic and pathologic criteria determine the success of therapies for lung cancer (i.e., Response Evaluation Criteria in Solid Tumors (RECIST), TNM classification) [31]. Problems such as the lack of clinical or sonographical skills as well as use of different examination methods, such as contrast and non-contrast enhanced tomography, may impede adequate interpretation of therapy response. Histopathology provides high sensitivity and specificity in tissue analysis. However, sample collection involves extensive risks by invasive techniques and is limited by the heterogeneity of tumor tissue that may result in false negative results and require resampling [32]. In addition, a biochemical response to chemotherapy is difficult to detect and evaluate by imaging techniques [33]. Determination of circulating tumor markers to evaluate therapy response and the course of disease is easy and cost-efficient to perform [34]. For this purpose, evaluation of new biomarkers for routine diagnostics is needed to optimize clinical decision making on therapy options and disease-management during and after treatment. To achieve this, we evaluated the relevance of soluble HMGB1 in serum samples for the prediction and monitoring of therapy response as well as the estimation of prognosis in patients with advanced lung cancer.

## 2. Patients, Materials and Methods

In a retrospective study, HMGB1 levels were assessed by ELISA in the sera of 96 patients with newly diagnosed or recurrent advanced lung cancer (79 NSCLC; 14 small-cell lung carcinoma (SCLC), 3 Mesothelioma). For higher homogeneity in subsequent analyses; NSCLC and SCLC patients were evaluated separately; patients with mesothelioma were not taken into consideration due to the limited sample number. Modal histopathology in NSCLC was adenocarcinoma in 30 patients (38.0%), squamous cell carcinoma in 26 patients (32.9%), large cell cancer in 6 patients (7.6%) and NSCLC not further specified in 17 patients (21.5%). Overall age distribution ranged between 38 and 83 years (medians NSCLC and SCLC, 61 years). The NSCLC cohort consisted of 26 female and 53 male patients and the SCLC of 6 female and 8 male patients who underwent chemotherapy at the Asklepios-Lungenfachklinik Gauting between January 1999 and June 2002. The most prevalent chemotherapy regimen in NSCLC patients was gemcitabine/cisplatin (N = 20, 25%), followed by other gemcitabine-based combinations (N = 18, 23%), mitomycin-based regimens (N = 16, 20%), docetaxel (N = 6, 8%), taxol (N = 4, 5%), ifosfamide-based therapy (N = 3, 4%), cisplatin (N = 2, 3%) and other regimens (N = 10, 13%). Patients with SCLC were treated under CEV regimen (cisplatin/etoposide/vincristine, N = 6, 43%), topotecan (N = 5, 36%), ifosfamide/adriamycin (N = 2, 14%) and cisplatin (N = 1, 7%). Patients suffering from mesothelioma were treated with cisplatin/multitargeted antifolate (MTA) (N = 2, 67%) or gemcitabine (N = 1, 33%). 

Before chemotherapy, 5 patients underwent surgical treatment, 3 received surgical treatment and adjuvant radiation, 3 mesothelioma patients underwent pleurodesis and 1 patient received neoadjuvant radiochemotherapy. Before initializing chemotherapy, every patient was evaluated by whole-body computed tomography (CT), bone scintigraphy and bronchoscopy with biopsy. Therapy response was evaluated by CT-based staging after chemotherapy cycle 2 according to WHO classification with “partial remission“ being defined as a tumor reduction ≥ 50%, “progression” as tumor growth ≥ 25% or appearance of a new lesion and “No change“ as status in which tumor reduction was ˂ 50% or tumor growth < 25% [35,36]. If the response was “no change”, an additional CT-based staging was performed after cycle 4. Patients with partial remission or no change were considered assigned to the group of patients with “favourable therapy response”, whereas patients with tumor progression at this additional time point were assigned to the group of patients with “no response to therapy”. 

Blood samples were collected in clinical routine settings before chemotherapy as well as before cycles 2 and 3 in serum S-Monovette^®^ vials (Fa. Sarstedt, Nürmbrecht, Germany). After blood collection, samples were centrifuged, transferred into cryogenic vials and stored at −80 °C until measurement. In parallel, established tumor markers carcinoembryonic antigen (CEA), cytokeratin 19-fragments (CYFRA 21-1) and neuron specific enolase (NSE) were assessed for routine diagnostics on the Elecsys Cobas E411 immunoanalyzer (Roche Diagnostics, Mannheim, Germany) in the Department of Laboratory Medicine at the Lungenfachklinik Gauting. Subsequently, HMGB1 sera levels were measured by an enzyme-linked immunosorbent assay (ELISA, Catalog# ST51011; IBL; Hamburg, Germany) in the Institute of Clinical Chemistry and Clinical Pharmacology at the University Hospital Bonn. The assay is based on a classic sandwich-enzyme-linked immunosorbent assay principle for the quantitative determination of HMGB1 in human serum and plasma. Briefly, wells in microtitre plates were coated with immobilized purified and polyclonal anti-HMGB1-antibodies that specifically bind serum-HMGB1. Another enzyme-linked (peroxidase) antibody functioned as detector and was also capable of specific HMGB1 binding. After reaction of peroxidase with 3,3,5,5′-Tetramethylbenzidine (TMB), photometric detection was done at 450 nm and HMGB1 concentrations were calculated by use of an appropriate calibration curve. 

Data analyses and the distribution of values are illustrated in tables and diagrams. Medians, interquartile ranges (IQR) and ranges are listed in tables for the subgroups. Distribution of values is demonstrated in box plot graphics. Differences between the various groups were calculated by Mann–Whitney U or χ^2^ tests, according to the scale of measure. Spearman’s rank-order correlation ϱ was used for correlation analysis. Diagnostic power of the single markers is shown by receiver operating characteristic (ROC) curves. Areas under the curves (AUC) and sensitivities at 90% and 95% specificity versus control groups were calculated. For monitoring purposes, HMGB1 baseline values before the first, second and third cycle and the percental changes between cycles 1–2 and cycles 1–3 were considered for statistical analysis. Prognostic relevance of HMGB1 and established tumor markers were univariately tested by log-rank tests and are illustrated by Kaplan–Meier curves. In general, *p*-values <0.05 were considered statistically significant. Concerning the explorative character of the data analysis, *p*-values were not adjusted. Summary, editing and analysis of data were performed with Microsoft Office Professional Plus 2016(Word, Excel, Power Point, version 2101/build 13628.20308, Microsoft Corporation, Redmond, WA, USA) and IBM SPSS Statistics (version 23, International Business Machines Corporation, Armonk, NY, USA).

## 3. Results

As shown in Table 1, no significant differences in the basic and clinical characteristics (age and gender) were seen. About one third of the cohort were adenocarcinoma patients (32%), just under another third was represented by and squamous cell cancer patients (28%) while patients with SCLC (15%), large cell cancer (7%) and other NSCLC cancers (18%) were smaller groups. Most patients had metastasized disease (69%) and 29% were locally advanced. In total, 55% responded well to therapy (remission or no change), while 45% were progressive under therapy at radiologic staging after cycle 2 and 4.

**Figure 1:**
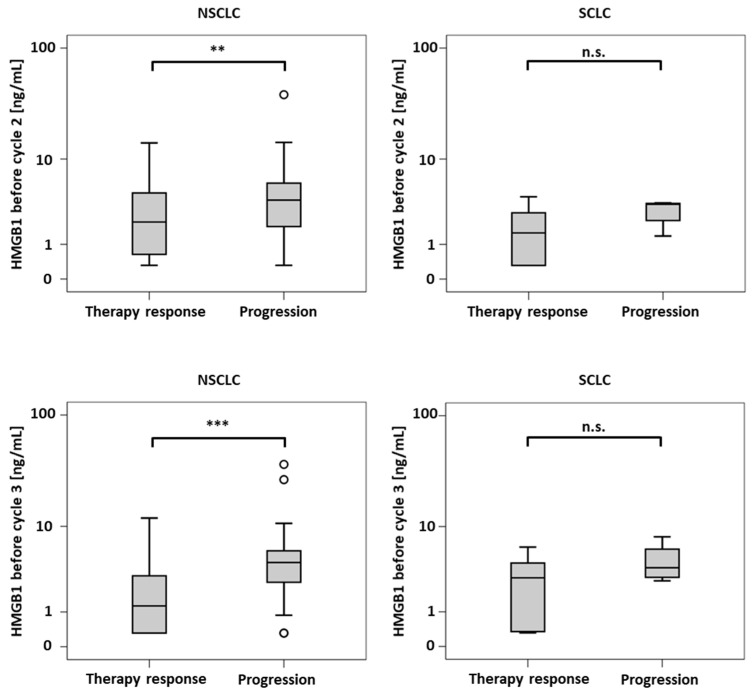
Boxplots of concentrations of high mobility group box 1 protein (HMGB1) before the second (top) and third therapy cycle (bottom) for non-small-cell lung carcinoma (NSCLC) (left) and small-cell lung carcinoma (SCLC) patients (right). n.s.: not significant, **: *p* < 0.01, ***: *p* < 0.001.

Biomarker levels in the diverse response groups of NSCLC and SCLC patients are shown in Table 2 and Table 3, respectively (Table 2 and Table 3). In NSCLC, the pretherapeutic levels of HMGB1, CEA and CYFRA 21-1 were not able to discriminate between response groups. However, both prior to cycles 2 and 3, levels of HMGB1 and CYFRA 21-1 were significantly lower in responsive patients than in progressive patients (see also Figure 1). In addition, HMGB1 and CYFRA 21-1 levels decreased significantly more from cycle one to two in patients who responded to therapy (to 63% and 61%, respectively) in comparison with non-responsive patients (remaining more or less stable at 106% and 98%, respectively). For CEA, no difference between the response groups was observed (Table 2).

In the SCLC subgroup, HMGB1 levels could not discriminate between responders and non-responders concerning both absolute values and kinetics. However, a trend could be noticed in the form of more pronounced changes from cycle 1 to cycle 2 (a decrease to 38% in responders vs. stable values at 108% in non-responders). This difference was however not found to be significant (*p* = 0.054), but this could be due to the small patient number this was not significant (*p* = 0.054). In contrast, CEA and NSE exhibited significantly lower absolute values at cycles 2 and 3 and stronger decreases from cycle 1 to 3 for patients with therapy response when compared with progressive patients who had higher values and increasing kinetics (Table 3, Figure 1).

**Figure 2:**
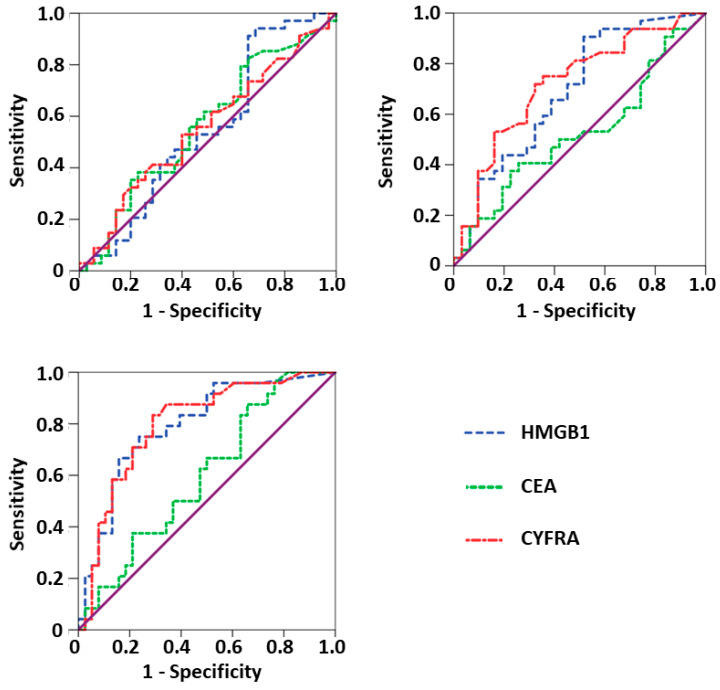
Receiver Operating Characteristic (ROC) curves for prediction/detection of progressive disease of non-small-cell lung carcinoma (NSCLC) patients calculated for high mobility group box 1 protein (HMGB1), carcinoembryonic antigen (CEA) and cytokeratin 19-fragments (CYFRA 21-1) in NSCLC patients a) before start of therapy (upper part left), b) prior to the second chemotherapy cycle (upper part right), and c) prior to the third chemotherapy cycle (lower part left).

ROC curves for the detection of progressive disease were calculated for the NSCLC group as this represented the largest group. Thereby, the sensitivity for detection of progression was tested against the specificity (patients with remission and stable disease as control group) for the whole range of possible cutoffs (Table 4 and Figure 2). Once again, HMGB1 and CYFRA 21-1 yielded significant results for AUCs of ROC curve analyses prior to cycle 2 (HMGB1: AUC = 0.690, *p* = 0.010; CYFRA 21-1: AUC = 0.719, *p* = 0.003) and prior to cycle 3 (HMGB1: AUC = 0.794, *p* < 0.001; CYFRA 21-1: AUC = 0.799, *p* < 0.001), with higher concentrations indicating failure of therapy. Sensitivities of HMGB1 at cycles 2 and 3 for a given specificity of 90% were 34.4% and 37.5%, respectively. Accordingly, sensitivities of 37.5% and 41.7% were calculated for CYFRA 21-1.

**Figure 3:**
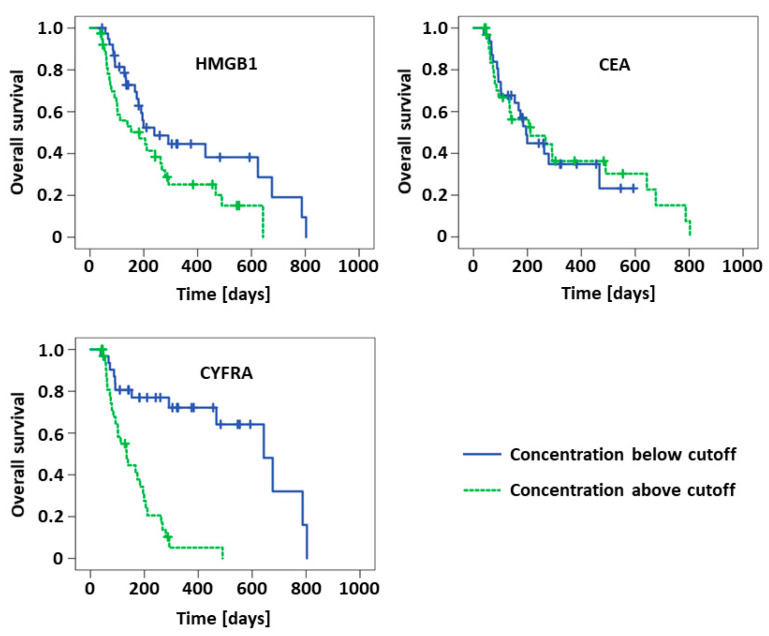
Kaplan–Meier curves of NSCLC patients possessing concentrations of HMGB1, CEA and CYFRA below or above the respective cutoff values at the second chemotherapy cycle.

**Figure 4:**
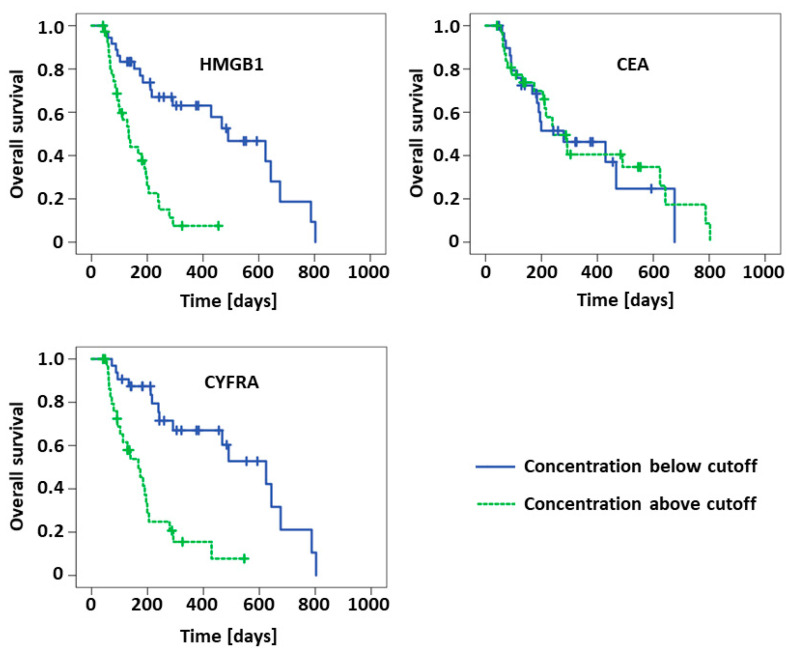
Kaplan–Meier curves of NSCLC patients possessing concentrations of high mobility group box 1 protein (HMGB1), carcinoembryonic antigen (CEA) and cytokeratin 19-fragments (CYFRA) below or above the respective cutoff values at the third chemotherapy cycle.

For evaluation of the prognostic value of the biomarkers in NSCLC patients, Kaplan–Meier curves and Log-Rank analyses for overall survival were used. Median concentrations of the respective biomarkers at the respective time points in the NSCLC cohort were applied as cutoff values for these analyses (*cf.* Table 2). Detailed results are given in Table 5 and visualized in Figure 3 and Figure 4, respectively. Cutoff values for biomarker median levels at the various treatment cycles were quite stable and varied only between 2.5 and 2.8 ng/mL for HMGB1, between 5.5 and 6.0 ng/mL for CEA and between 3.9 and 4.9 ng/mL for CYFRA 21-1. In accordance with the aforementioned results, concentrations below the cutoff concentrations of HMGB1 and CYFRA 21-1 at cycle 2 resulted in significantly longer median overall survival (HMGB1: 239 vs. 184 d, *p* = 0.038; CYFRA 21-1: 643 vs. 134 d, *p* > 0.001). The absolute difference in survival times was even higher at cycle 3 for HMGB1 (490 vs. 134 d, *p* < 0.001) and similar for CYFRA 21-1 (624 vs. 167 d, *p* < 0.001). While pretherapeutic CYFRA 21-1 also exhibited a significant difference concerning survival (291 vs. 139 d, *p* = 0.001), there was no prognostic value of pretherapeutic HMGB1 observed at this time point. CEA was not useful for prognostic estimation either.

Further subgroup analyses were performed for the predominant histological subtypes adenocarcinomas and squamous cell carcinomas (figures and tables in Supplementary Materials). In adenocarcinomas, HMGB1 was higher in non-responders and differed significantly between responders and non-responders before cycles 2 and 3 as well as between cycles 1 and 2. In squamous cell carcinomas, HMGB1 only differed between responders and non-responders before cycle 3, with responders possessing a higher median concentration (5.02 ng/mL vs. 2.10 ng/mL). ROC analyses for the detection of progressive disease yielded significant AUCs for HMGB1 concentrations in adenocarcinomas before cycles 2 (AUC = 0.764, *p* = 0.015) and 3 (AUC = 0.896, *p* < 0.001), whereas in squamous cell carcinomas, a significant differentiation by HMGB1 levels was only achieved before cycle 3 (AUC = 0.879, *p* = 0.002). In Kaplan–Meier curves and Log-Rank tests with median concentrations of HMGB1 in the respective subgroup and at the respective time points as cutoff values, a significant stratification between high and low concentrations was found only in adenocarcinomas when median HMGB1 concentrations before therapy (*p* = 0.038) or before cycle 2 (*p* = 0.025) were applied. In both cases, higher concentrations of HMGB1 were associated with shorter overall survival.

## 4. Discussion

HMGB1, as a characteristic danger associated molecular pattern (DAMP) marker, was found to be elevated intracellularly and released into bodily fluids in various diseases, including poorly differentiated neoplasms [25]. Naumnik et al. reported on elevated pretherapeutic levels of HMGB1 in 40 patients with NSCLC, but—possibly due to the low number of patients—there was no significant differentiation between stages IIIb und IV nor any prognostic information obtained by HMGB1 [37]. The authors concluded that HMGB1 (together with survivin and VEGF) had no clinical impact concerning prognosis or survival times. In another large study comprising 145 NSCLC patients, cancer patients had significantly higher HMGB1 levels when compared to 77 patients with chronic obstructive lung disease and 49 healthy individuals. Furthermore, HMGB1 levels correlated with both the stage of disease and tumor size, and showed decreased levels after resection of the tumor [38]. A meta-analysis and literature review investigating the role of HMGB1 in NSCLC concluded that the concentration of HMGB1 was elevated in both the lung tissue and serum samples of NSCLC patients, and it was suggested that HMGB1 may serve as a diagnostic biomarker in NSCLC. [39]

Similar results were described for other tumors such as gastric cancer [18], hepatocellular cancer [40], metastatic colorectal cancer [41,42], cervical cancer [43], breast cancer [44] and advanced pancreatic cancer [45]. Concerning the ever-rising worldwide burden of lung cancer and poor therapy outcome due to late diagnoses in advanced stages of the disease, new diagnostic approaches are necessary to ensure timely interventions with appropriate treatments. Circulating biomarkers offer multiple advantages as they are easily obtained, are cost-efficient and reflect dynamic therapy response [46]. However, due to the non-tumor specific nature of HMGB1 and the high concentrations thereof, which are also found in the serum and plasma of patients with other acute pathologies such as sepsis and trauma [44,47], the use of HMGB1 for tumor diagnosis is questionable. Nevertheless, it could bear important information for the monitoring of cytostatic therapies as well as for the estimation of prognostic outcome of cancer patients [44,47].

Therefore, we analyzed soluble HMGB1 with regard to its relevance for prediction of therapy response and prognosis in advanced lung cancer patients and compared it with established lung cancer tumor markers CYFRA 21-1, CEA and NSE to investigate whether HMGB1 was a superior marker or whether it could add value to the information of other markers already used in routine diagnostics. While targeted and immune therapies are often applied in lung cancer nowadays, many patients still receive classical chemotherapy protocols such as those used in this retrospective study–especially if the molecular tumor status does not clearly indicate one of the innovative therapy types [48,49].

Interestingly, pretherapeutic levels of HMGB1 and other tumor markers were not predictive for therapy response in NSCLC patients. This contradicts findings by Jakubowska et al., who found higher HMGB1 levels prior to therapy to be linked to poor prognosis [50]. However, in our study HMGB1 and CYFRA 21-1 levels prior to cycles 2 and 3 showed great differences between response groups with lower concentrations and steeper decreases in serial determinations in patients with remission or stable disease as compared with progressive patients. This means that before the start of therapies, all patients had a similar biochemical status (regarding the markers investigated here) and changes in marker levels were clearly influenced by effective therapies. This is all the more remarkable since different pathophysiological processes are represented by each of the different markers. HMGB1 as a classical DAMP has been linked to i) proinflammatory conditions in the microenvironment of the tumor that are part of the extended hallmarks of cancer [51], ii) to general inflammatory reactions within the body created by the tumor itself, and iii) regarded as a consequence of therapy side effects. In contrast, cytokeratin 19 fragments (CYFRA 21-1) are classical tumor-associated antigens that are mainly expressed in lung tissue and released from advanced cancers as a reflection of high tumor activity or large tumor mass. In addition, CYFRA 21-1 was also described as a marker of apoptotic cell death. The decline of both markers during treatment can be interpreted as amelioration in terms of less tumor activity, shrinking mass and less inflammatory reactions within the body. In this respect, it has to be considered that the half-life times of both markers are quite short, i.e., in the order of hours (CYFRA 21-1) or few days (HMGB1) and that the second cycle was applied only after an interval of three weeks. This means that the measured values rather reflect the total situation of the disease than the immediate effects of the therapy on the marker levels.

In accordance with these findings, the analytical performance parameters for the detection of progressive disease showed comparably good results for HMGB1 and CYFRA 21-1 prior to cycles 2 and 3 in NSCLC patients. Moreover, sensitivities at given specificities of 90% were comparably good at both time points indicating that 30% to 40% of non-responding patients could be identified only on the basis of biomarker analyses with a high specificity of 90%. Although there is still room for improvement, it is clear that a subgroup of patients could benefit from serial biomarker assessments for earlier detection of progressive disease while undergoing therapy. This will facilitate timely interventions by medical teams to make changes to the therapy strategies and to select more promising ones. The pan-cancer marker CEA, which has repeatedly been shown to be a valuable parameter in lung cancer monitoring [13,46,52], was not predictive in our setting.

Beyond therapy monitoring, prognostic estimation of overall survival is crucial for patients with advanced cancer. Patients may suffer from only temporary treatment efficacy, early tumor recurrence or progression after therapy, severe side effects, sepsis, organ failure etc. that greatly determine their lifespan and quality of life. With respect to biomarkers, CYFRA 21-1 was the strongest prognostic parameter at all time points investigated. Low levels of CYFRA 21-1 always correlated with highly significant longer median survival. For HMGB1, slightly significant prognostic value was seen before cycle 2, but a strong difference between prognostic groups was observed prior to cycle 3. This shows that the therapeutic impact of therapy is not reflected in HMGB1 concentrations until cycle 3. On the other hand, levels of tumor-associated CYFRA 21-1 already indicated later prognosis before the beginning of the therapy. 

The results obtained in this study strongly suggest a potential value of HMGB1 in NSCLC. Compared to the known NSCLC biomarker CYFRA 21-1, HMGB1 demonstrated similar diagnostic value in terms of ROC curves and comparable prognostic value with respect to stratification of overall survival using Kaplan–Meier curves. Our results are consistent with other studies performed on patients with gastric cancer [18], cervical cancer [43] und hepatically metastasized colorectal cancer [41,42], in which HMGB1 showed prognostic value as well. Furthermore, a recent meta-analysis investigated the role of HMGB1 in estimating overall and progression-free survival of patients with diverse cancers [53]. Depending on the type of cancer, investigated subgroups, geographical distribution, sample size and detection method, the authors reported hazard ratios for overall survival between 1.54 and 2.93, indicating longer overall survival in patients with low concentrations of HMGB1. Similarly, the prognostic value of CYFRA 21-1 has been analyzed and demonstrated by numerous studies for patients with lung cancer (summarized in [13]). In particular, a pooled analysis of more than 2000 patients has revealed the reproducible and strong prognostic information of CYFRA 21-1 in patients with early and late stage lung cancers [54]. Meanwhile, the high relevance of CYFRA 21-1 and CEA for monitoring the therapy response of advanced NSCLC patients was also demonstrated by a comprehensive meta-analysis [55]. Despite considerable heterogeneity in study designs, marker assessment and interpretation, outcome definition etc., the clinically relevant and often reproducible predictive value of CYFRA 21-1 (and also CEA) could be seen in most studies included. Several recent developments have been aimed at establishing algorithms for the calculation of predictive and prognostic scores that show promising results in early and late stage disease [56,57]. It will be interesting to see whether HMGB1 as a marker with different pathophysiological background will add to these established markers. 

Despite the low number of patients with SCLC, significant results for therapy monitoring were also found for CEA and NSE prior to cycles 2 and 3, which certainly would have to be confirmed in larger patient cohorts.

Although the results of the present study are noteworthy, there are some limitations of the study such as the retrospective character, the inclusion of larger (NSCLC) and smaller subgroups (SCLC), the classical chemotherapeutic treatment, the long storage time until HMGB1 measurement, and the use of a non-diagnostic immunoassay (RUO-test). On the other hand, the ELISA assay was validated analytically and tested for precision with preanalytical influences [58] and has been used in diverse clinical biomarker studies in the past [47,59,60,61]. Samples were analyzed in a standardized procedure regarding collection, storage, treatment prior to measurement and analysis. High quality standards were obtained by standardized calibration curves, internal and external controls, measurement of serial samples within the same runs and final crossplate checks to minimize inter-assay variations. Radiological imaging analyses were performed by experienced, independent radiologists with identical methods and data interpretation was done independently from data acquisition. 

Another limitation is the lack of an independent validation cohort. However, such validation is beyond the scope of this work. Although it is one of the largest studies concerning the potential of HMGB1 as a predictive and monitoring biomarker in lung cancer, the number of patients was still deemed too low to sensibly split the cohort into a discovery and validation sample while keeping statistical power at an appropriate level. The study was designed as an explorative investigation, and the results would need to be validated in a prospective study with an even larger sample. Nevertheless, this is a well-designed study investigating HMGB1 in serial serum samples of lung cancer patients at defined time points prior to chemotherapy cycles 1, 2, and 3 and correlating the results with the response to therapy and prognostic outcome of the patients as well as comparing the new marker HMGB1 with established lung cancer tumor markers CEA, CYFRA 21-1 and NSE. In doing so, we found notable results in support of HMGB1 as a predictive and prognostic marker, thereby demonstrating the potential of HMGB1 as a promising target for further investigations in prospective studies, both as a single biomarker or as part of a multimarker panel for diagnostic and prognostic improvements in NSCLC.

## 5. Conclusions

The present study unveiled soluble HMGB1 as a new and highly valuable biomarker for the prediction and monitoring of the therapy response and prognosis in advanced NSCLC patients. It displayed results comparable to the lung tumor associated antigen CYFRA 21-1. Further studies will have to confirm these promising findings and show whether HMGB1 as a classical DAMP has additive value to established tumor markers.

## Figures and Tables

**Table 1:** Characterization of study cohort. NSCLC: non-small-cell lung carcinoma, SCLC: small-cell lung carcinoma, UICC: union international contre le cancer.

	Total	NSCLC	SCLC	*p*-Value
N	93	79	14	
**Age/gender**				
Age (years) (mean ± SD)	61.1 ± 10.0	61.1 ± 10.6	61.0 ± 6.2	0.776
Female, N (%)	32 (34.4)	26 (32.9)	6 (42.9)	0.546
Male, N (%)	61 (65.6)	53 (67.1)	8 (57.1)	
**Histology, N (%)**				
Large cell cancer	6 (6.5)	6 (7.6)		
Adenocarcinoma	30 (32.3)	30 (38.0)		
Squamous cell carcinoma	26 (28.0)	26 (32.9)		
Other NSCLC	17 (18.3)	17 (21.5)		
SCLC	14 (15.1)		14 (100)	
**Stage, N (%)**				
UICC stage 3	27 (29.0)	21 (26.6)	6 (42.9)	
UICC stage 4	64 (68.8)	56 (70.9)	8 (57.1)	
No staging available	2 (2.2)	2 (2.5)	-	
**Therapy response, N (%)**				
Remission/no change	51 (54.8)	41 (51.9)	10 (71.4)	
Progression	42 (45.2)	38 (48.1)	4 (28.6)	
**Overall survival**				
Mean (days)		224	293	

**Table 2:** Prediction of therapy response in NSCLC by biomarkers.

NSCLC	Response	N	Median	IQR	Range	*p*-Value
**Pretherapeutic**						
HMGB1	R NR	41 38	2.84 2.84	3.9 3.6	0.3–13.4 0.3–13.2	0.641
CEA	R NR	35 34	5.10 5.85	9.6 26.2	1.7–2299 1.6–775	0.371
CYFRA 21-1	R NR	35 34	4.40 6.65	8.2 10.0	0.0–67.0 0.3–72.0	0.453
**Cycle 2**						
HMGB1	R NR	40 38	2.12 3.84	4.0 4.1	0.3–14.2 0.3–38.8	**0.009**
CEA	R NR	32 32	5.55 5.75	10.3 21.7	1.6–279 1.6–647	0.663
CYFRA 21-1	R NR	33 32	2.30 6.25	4.0 10.2	0.3–51.6 1.0–54.0	**0.006**
**Cycle 3**						
HMGB1	R NR	40 34	1.25 4.35	2.9 3.4	0.3–12.0 0.3–36.7	**<0.001**
CEA	R NR	39 24	5.30 6.90	9.9 15.7	1.4–2400 2.7–393	0.188
CYFRA 21-1	R NR	38 26	2.30 8.60	3.5 13.3	0.5–92.7 1.1–68.1	**<0.001**
**Changes Cycles 1–2 (%)**						
HMGB1	R NR	41 38	62.8 106	87.8 119	0.0–1764 9.4–2446	**0.008**
CEA	R NR	35 34	85.7 97.4	57.4 44.6	0.0–290 0.0–437	*0.086*
CYFRA 21-1	R NR	34 34	61.2 97.9	59.0 120	0.0–305 0.0–500	**0.001**
**Changes Cycles 1–3 (%)**						
HMGB1	R NR	41 38	50.2 84.3	103 151	0.0–1096 0.0–2317	*0.058*
CEA	R NR	35 34	92.3 89.2	71.1 138	0.0–340 0.0–702	0.621
CYFRA 21-1	R NR	34 34	53.8 79.0	63.1 178	0.0–461 0.0–1833	0.213

Median concentrations, ranges and comparison between responders (R = remission and no change; upper rows) and non-responders (NR = progression; lower rows) of investigated biomarkers in NSCLC patients. Concentrations are given in ng/mL. IQR: interquartile range, HMGB1: high mobility group box 1 protein, CEA: carcinoembryonic antigen, CYFRA 21-1: cytokeratin 19-fragments. *p*-values in bold indicate statistically significant results, *p*-values in italics indicate statistical trends (*p* < 0.10).

**Table 3:** Prediction of therapy response in SCLC by biomarkers.

SCLC	Response	N	Median	IQR	Range	*p*-Value
**Pretherapeutic**						
HMGB1	R NR	10 4	2.84 3.29	4.6 3.4	1.2–8.9 0.3–4.5	0.839
CEA	R NR	9 3	3.90 12.6	6.7 -	1.9–10.9 4.6–93.4	*0.064*
NSE	R NR	8 4	18.7 33.6	64.7 91.4	8.7–591 18.3–133	0.283
**Cycle 2**						
HMGB1	R NR	10 4	1.54 3.44	2.5 1.7	0.3–4.2 1.4–3.6	0.106
CEA	R NR	8 3	5.65 47.7	6.1 -	1.8–12.4 7.3–94.1	**0.048**
NSE	R NR	9 3	11.9 35.5	4.4 -	8.9–24.2 34.2–39.6	**0.009**
**Cycle 3**						
HMGB1	R NR	10 4	2.95 3.87	4.2 4.2	0.3–6.3 2.7–8.0	0.304
CEA	R NR	9 4	4.70 28.5	5.2 111	1.5–23.5 6.7–141	**0.020**
NSE	R NR	8 4	12.6 60.7	4.4 55.0	8.0–15.9 38.4–97.8	**0.004**
**Changes Cycles 1–2 (%)**						
HMGB1	R NR	10 4	38.0 108	74.6 277	8.6–218 79.8–437	*0.054*
CEA	R NR	9 3	94.7 101	88.0 -	0.0–154 0.0–379	0.727
NSE	R NR	8 4	63.9 127	92.7 163	0.0–122 0.0–187	0.368
**Changes Cycles 1–3 (%)**						
HMGB1	R NR	10 4	72.1 206	111 643	19.2–254 72.9–870	0.106
CEA	R NR	9 3	94.7 151	66.7 -	0.0–230 146–387	**0.036**
NSE	R NR	8 4	54.2 152	71.5 245	0.0–120 73.5–356	**0.028**

Median concentrations, ranges and comparison between responders (R = remission and no change; upper rows) and non-responders (NR = progression; lower rows) of investigated biomarkers in SCLC patients. Concentrations are given in ng/mL. HMGB1: high mobility group box 1 protein, CEA: carcinoembryonic antigen, CYFRA 21-1: cytokeratin 19-fragments. *p*-values in bold indicate statistically significant results, *p*-values in italics indicate statistical trends (*p* < 0.10).

**Table 4:** Performance of biomarkers for prediction of non-response to therapy. AUC: Area under the curve, HMGB1: high mobility group box 1 protein, CEA: carcinoembryonic antigen, CYFRA 21-1: cytokeratin 19-fragments. AUCs and *p*-values in bold indicate statistically significant results.

	AUC	95% CI	Sensitivity at 90% Specificity	Sensitivity at 95% Specificity	*p*-Value
**Pretherapeutic**					
HMGB1	0.549	0.410–0.687	3.4%	2.0%	0.486
CEA	0.563	0.426–0.699	11.4%	2.9%	0.371
CYFRA 21-1	0.553	0.416–0.689	14.3%	2.9%	0.453
**Cycle 2**					
HMGB1	**0.690**	0.558–0.821	34.4%	6.3%	**0.010**
CEA	0.523	0.378–0.667	18.8%	6.3%	0.757
CYFRA 21-1	**0.719**	0.592–0.847	37.5%	15.6%	**0.003**
**Cycle 3**					
HMGB1	**0.794**	0.680–0.909	37.5%	20.8%	**<0.001**
CEA	0.598	0.456–0.739	16.7%	8.3%	0.198
CYFRA 21-1	**0.799**	0.686–0.913	41.7%	4.2%	**<0.001**

Calculation of Receiver Operating Characteristic (ROC) curve analyses show sensitivity and specificity for prediction/detection of progressive disease of NSCLC patients over the whole spectrum of possible cutoff values. Performance criteria are the area under the curve (AUC) and the sensitivities at specificities of 90% and 95%. *p*-values in bold indicate statistically significant results.

**Table 5:** Performance of biomarkers for prognosis of overall survival.

		Below Cutoff		Above Cutoff		
	Cutoff	Median OS (d)	95% CI	Median OS (d)	95% CI	*p*-Value
Pretherapeutic						
HMGB1	2.84	195	116–274	239	165–313	0.742
CEA	5.50	180	140–220	211	140–282	0.896
CYFRA 21-1	4.90	291	10–572	139	73–205	**0.001**
Prior to cycle 2						
HMGB1	2.52	239	89–389	184	51–317	**0.038**
CEA	5.60	195	147–233	211	12–410	0.888
CYFRA 21-1	4.40	643	438–848	134	86–182	**<0.001**
Prior to cycle 3						
HMGB1	2.69	490	296–684	134	98–170	**<0.001**
CEA	6.00	279	30 -528	242	130–354	0.721
CYFRA 21-1	3.85	624	399–849	167	103–231	**<0.001**

Kaplan–Meier and Log-Rank analyses show of the prognostic value of biomarkers in NSCLC patients if median is used as cutoff. Median overall survival (OS) in days (d) with 95% confidence intervals (CI) is given for the group with values below and above the cutoff. Significance as calculated according Log-Rank analysis. Cutoffs are given in ng/mL. *p*-values in bold indicate statistically significant results. HMGB1: high mobility group box 1 protein, CEA: carcinoembryonic antigen, CYFRA 21-1: cytokeratin 19-fragments.

## Data Availability

Raw data of this study can be made available on request.

## Supplementary Materials

The following are available online at https://www.mdpi.com/2075-4418/11/2/356/s1.
