# Supplementary material for: Soluble High Mobility Group Box 1 (HMGB1) Is a Promising Biomarker for Prediction of Therapy Response and Prognosis in Advanced Lung Cancer Patients
Source: Diagnostics (Basel). 2021 Feb 20;11(2):356. doi: 10.3390/diagnostics11020356 (PMC7924191; doi:10.3390/diagnostics11020356)
Supplement: Supplementary file 1 [file diagnostics-11-00356-s001.pdf]

Supporting Information to „Soluble High Mobility Group Box 1 (HMGB1) is a Highly Valuable Biomarker for Prediction of Therapy Response and Prognosis in Advanced Lung Cancer Patients"

Nikolaus A. Handke, Alexander Rupp, Nicolai Trimpop, Joachim von Pawel, Stefan Holdenrieder

1 Subgroup analyses of HMGB1 in adenocarcinomas and squamous cell carcinomas

1.1 Concentrations of HMGB1 at the different time points

Table 1: HMGB1 concentrations in patients with adenocarcinomas.

Time point	Response	N	Median	IQR	Range	p-Value
Pretherapeutic	R	16	2.64	2.5	0.3 – 10.6	0.448
	NR	14	3.69	2.9	0.5 – 8.3	
Cycle 2	R	15	1.20	2.6	0.3 – 4.6	**0.014**
	NR	14	3.39	3.6	0.3 – 38.8	
Cycle 3	R	16	0.57	1.3	0.3 – 3.6	**< 0.001**
	NR	12	4.25	4.1	0.9 – 36.7	
Changes Cycles 1-2 (%)	R	16	39.2	58.5	0 – 135.3	**0.002**
	NR	14	94.8	87.1	30.6 – 2446.3	
Changes Cycles 1-3 (%)	R	16	46.2	56.6	4.7 – 108.6	0.077
	NR	14	78.6	133.1	0 – 2317.0	

Table 2: HMGB1 concentrations in patients with squamous cell carcinomas.

Time point	Response	N	Median	IQR	Range	p-Value
Pretherapeutic	R	15	2.84	3.2	0.3 – 13.4	0.760
	NR	11	2.47	0.8	0.9 – 11.0	
Cycle 2	R	15	2.11	4.1	0.3 – 6.6	0.097
	NR	11	3.22	4.7	1.2 – 14.3	
Cycle 3	R	14	2.10	3.1	0.3 – 7.2	**0.001**
	NR	10	5.02	3.7	2.8 – 26.9	
Changes Cycles 1-2 (%)	R	15	93.9	106.7	3.8 – 258.2	0.305
	NR	11	130.6	184.7	15.8 – 508.7	
Changes Cycles 1-3 (%)	R	15	50.9	168.1	0 – 636.7	0.069
	NR	11	186.1	492.1	0 – 952.9	

Patients with adenocarcinomas did not differ from patients with squamous cell carcinomas in terms of response to therapy according to a χ^2 test ($\chi^2 = 0.107$, $p = 0.793$).

1.2 ROC curve analyses

Table 3: Performance of HMGB1 for prediction of non-response to therapy in the subgroups of adenocarcinomas and squamous cell carcinomas. Calculation of Receiver Operating Characteristic (ROC) curve analyses show sensitivity and

specificity for prediction / detection of progressive disease of NSCLC patients over the whole spectrum of possible cutoff values. Performance criteria are the area under the curve (AUC) and the sensitivities at specificities of 90% and 95%.

Time point	AUC	95% CI	Sensitivity at 90% Specificity	Sensitivity at 95% Specificity	p-value
**Pretherapeutic**					
Adeno	0.585	0.374 – 0.796	0.000	0.000	0.430
Squamous cell	0.461	0.232 – 0.689	0.091	0.000	0.736
**Cycle 2**					
Adeno	0.764	0.590 – 0.939	0.357	0.357	**0.015**
Squamous cell	0.697	0.494 – 0.900	0.364	0.091	0.092
**Cycle 3**					
Adeno	0.896	0.779 – 1.000	0.750	0.583	**< 0.001**
Squamous cell	0.879	0.739 – 1.000	0.400	0.300	**0.002**

1.3 Survival analyses

Median concentrations of the respective subgroup at the respective time point were used as cutoff values in Kaplan-Meier analyses.

Table 4: Performance of HMGB1 in adenocarcinomas and squamous cell carcinomas for prognosis of overall survival. Kaplan-Meier and Log-Rank analyses show of the prognostic value of biomarkers in NSCLC patients if median is used as cutoff. Median overall survival (OS) in days (d) with 95% confidence intervals (CI) is given for the group with values below and above the cutoff. P-values were calculated according to Log-Rank analyses.

		Below cutoff		Above cutoff		
	**Cutoff**	**Median OS (d)**	**95% CI**	**Median OS (d)**	**95% CI**	**p-value**
**Pretherapeutic**						
Adeno	2.76	624	0 – 1390	66	82 – 340	**0.038**
Squamous cell	2.56	190	50 – 330	292	130 – 454	0.390
**Prior to cycle 2**						
Adeno	2.35	429	112 – 746	75	0 – 282	**0.025**
Squamous cell	2.59	190	168 – 212	267	129 – 405	0.880
**Prior to cycle 3**						
Adeno	1.35	624	0 – 1268	62	78 – 320	0.167
Squamous cell	3.03	267	0 – 542	180	104 – 256	0.072
